# Determinants of Hair Manganese, Lead, Cadmium and Arsenic Levels in Environmentally Exposed Children

**DOI:** 10.3390/toxics6020019

**Published:** 2018-03-22

**Authors:** Thomas Jursa, Cheryl R. Stein, Donald R. Smith

**Affiliations:** 1 Department of Microbiology and Environmental Toxicology, University of California, Santa Cruz, CA 95064, USA; tpjursa@ucsc.edu; 2 Department of Child and Adolescent Psychiatry, Hassenfeld Children’s Hospital at NYU Langone, Child Study Center, New York University, New York, NY 10016, USA; Cheryl.Stein@nyumc.org

**Keywords:** manganese, lead, cadmium, arsenic, hair, children, environment

## Abstract

Biomarkers of environmental metal exposure in children are important for elucidating exposure and health risk. While exposure biomarkers for As, Cd, and Pb are relatively well defined, there are not yet well-validated biomarkers of Mn exposure. Here, we measured hair Mn, Pb, Cd, and As levels in children from the Mid-Ohio Valley to determine within and between-subject predictors of hair metal levels. Occipital scalp hair was collected in 2009–2010 from 222 children aged 6–12 years (169 female, 53 male) participating in a study of chemical exposure and neurodevelopment in an industrial region of the Mid-Ohio Valley. Hair samples from females were divided into three two centimeter segments, while males provided a single segment. Hair was cleaned and processed in a trace metal clean laboratory, and analyzed for As, Cd, Mn, and Pb by magnetic sector inductively coupled plasma mass spectrometry. Hair Mn and Pb levels were comparable (median 0.11 and 0.15 µg/g, respectively) and were ~10-fold higher than hair Cd and As levels (0.007 and 0.018 µg/g, respectively). Hair metal levels were higher in males compared to females, and varied by ~100–1000-fold between all subjects, and substantially less (<40–70%) between segments within female subjects. Hair Mn, Pb, and Cd, but not As levels systematically increased by ~40–70% from the proximal to distal hair segments of females. There was a significant effect of season of hair sample collection on hair Mn, Pb, and Cd, but not As levels. Finally, hair metal levels reported here are ~2 to >10-fold lower than levels reported in other studies in children, most likely because of more rigorous hair cleaning methodology used in the present study, leading to lower levels of unresolved exogenous metal contamination of hair.

## 1. Introduction

Exposure biomarkers play an important role in estimating the internal dose of a person exposed to an environmental contaminant, and they are often essential in determining exposure–health effect relationships in epidemiological studies [1,2,3,4,5,6,7,8,9]. For example, lead (Pb) levels in blood and bone are accepted as well-validated Pb exposure biomarkers, and they helped establish the association between Pb exposure and health risk in children and adults [7,10,11]. Similarly, blood cadmium (Cd) levels have been shown to reflect Cd exposure from environmental sources [8,9]. However, for metals such as manganese (Mn), studies are mixed on whether blood Mn levels are an indicator of exposure and risk of health effects, presenting a need to develop and validate alternative exposure biomarkers [1,2,3]. Recent studies have suggested that hair Mn levels may help fill this need [2,3,12,13,14].

A number of studies have reported associations between levels of Mn in hair and Mn exposure and associated health effects in children and adolescents [12,13,14,15], including a recent review showing that hair was the most consistent and valid biomarker of Mn-associated health effects in children [3]. Similarly, levels of some other metals in hair, including mercury and arsenic, have been reported as both meaningful exposure biomarkers and indicators of health risks from exposure [16,17]. Hair may provide some practical advantages over other tissues as an exposure biomarker; scalp hair grows at a rate of roughly one centimeter per month, providing a possible indicator of exposure integrated over periods of one to five months or more, depending on the length of collected hair [18,19]. Moreover, analysis of sequential sections of hair may provide useful information on the temporal variability of exposure, although few studies have investigated whether sequential hair segments are useful for retrospective exposure assessment over the duration of hair growth [17]. 

However, the potential utility of hair as an exposure/effect biomarker is not without some challenges. Most notably, hair is susceptible to contamination from exogenous sources such as dust, water, and use of hair products [14,17,20]. Studies reporting hair metal levels as a biomarker of exposure vary widely in the type of method used to remove exogenous contamination from the hair, with methods varying from a simple water rinse to detergent and acid sonication [2,12,14,17,21,22,23,24,25]. Not surprisingly, reported hair metal levels vary widely by study, though it is unclear if this reflects differences in exposure versus differences in effectiveness of cleaning exogenous contamination. Studies have shown that metal levels in hair are derived largely from exogenous contamination, with the rigor of hair cleaning prior to analyses affecting the contribution of exogenous contamination to the measured hair metal levels [14,20,26]. 

Here, we determined levels of Mn, Pb, Cd and arsenic (As) in scalp hair samples from 222 male and female children aged 6–12 years living in the Mid-Ohio Valley. For 169 female subjects, hair samples were cut into sequential segments to determine the reproducibility of hair metal levels within the same subject, and the variation in hair metal levels over different seasons of growth. Hair samples were cleaned using a rigorous cleaning method shown to effectively remove exogenous metal contamination [14] and processed for analyses by inductively coupled plasma–mass spectrometry (ICP-MS).

## 2. Methods

### 2.1. Subjects

Hair samples were collected and processed from 222 subjects age 6–12 years (169 female, 53 male) recruited through the C8 Health Project Neurobehavioral Development Follow-up, which was investigating the neurodevelopmental health effects of perfluoroocatnoate (PFOA) exposure in southeastern Ohio and northwestern West Virginia. A detailed description of subject recruitment, as well as information on subject demographics, residence, and medical histories collected via maternal report at the time of the neurodevelopment follow-up study in 2009–2010 is provided elsewhere [27]. Mothers provided informed consent and children provided verbal assent; child and mother each received $50 for participation. The Mount Sinai Program for the Protection of Human Subjects and the Battelle Centers for Public Health Research & Evaluation Institutional Review Board approved all study procedures. Investigations were carried out following the rules of the Declaration of Helsinki of 1975 (https://www.wma.net/what-we-do/medical-ethics/declaration-of-helsinki/), revised in 2008. Relevant to the present study, this Ohio River Valley region also hosts the longest operating ferromanganese refinery in North America in Marietta, OH (Eramet Marietta, Inc., Marietta, OH, USA), and studies by others have reported elevated Mn exposures and associated health effects in children in the region [2,28].

Hair samples were collected proximal to the occipital lobe scalp and stored in zip top plastic bags at room temperature until analysis. Male subjects provided a single 2 cm segment of hair proximal to the scalp, while female subjects provided longer hair samples that were cut into sequential 2 cm segments (0–2, 2–4, 4–6 cm from the scalp), based on the overall length of the sample. A single 2 cm hair sample was analyzed for all male subjects (*n* = 53), since most males had hair too short to provide multiple segments. For females (*n* = 169), three sequential 2 cm hair segments were available for 159 subjects (referred to as proximal, medial, and distal 2 cm segments, relative to the scalp), two hair segments were available for *n* = 4 females (*n* = 3 for proximal and medial, *n* = 1 for proximal and distal), and one hair segment was available for *n* = 6 females (all proximal). 

### 2.2. Experimental

All cleaning and processing of hair samples was conducted in a HEPA filtered-air trace metal clean room, using acid-cleaned labware and ultrapure trace metal grade reagents. Individual hair segments/samples weighing 5–30 mg each were cleaned of exogenous metal contamination as described previously [14]. Briefly, samples were placed in acid-cleaned 5 mL polypropylene syringe tubes and sonicated (20 min) in 0.5% Triton, rinsed five-times with ultrapure Milli-Q water, sonicated (10 min) in 1 N trace metal grade nitric acid (Fisher Scientific, Santa Clara, CA, USA), rinsed with 1 N nitric acid, and rinsed five-times with Milli-Q water, and then dried at 65 °C for 48 h. Subsequently, samples were digested in 0.5 mL 15.7 N quartz-distilled nitric acid (Fisher Scientific, optima grade) at 80 °C for 6 h in a Class-100 HEPA filtered-air fume hood, and then diluted with 5 mL Milli-Q water. For analyses, 0.25 mL of digestate was transferred to an acid-cleaned polyethylene microfuge tube, diluted with 0.25 mL Milli-Q water, and centrifuged at 13,000× *g* for analysis. Rhodium and thallium were added to samples as internal standards, and samples analyzed for Mn, Pb, Cd, and As by magnetic sector inductively coupled plasma mass spectrometry (Thermo Element XR ICP-MS, Waltham, MA, USA), as described elsewhere [1,14]. Methane was added to the argon (Ar) carrier gas to minimize ArCl formation. ^208^Pb, ^111^Cd, and ^113^Cd were measured in low resolution, while ^55^Mn and ^75^Ar were measured in medium resolution. Typical analytical limits of detection (LOD’s) over five analytical runs were 0.0077, 0.0038, 0.0004, and 0.0018 ng/mL for Mn, Pb, Cd, and As, respectively. For metal levels below the analytical LOD, the LOD was multiplied by 0.5 and adjusted using the sample dilution factor and sample weight of processed hair to derive a value for half the procedural detection limit, and that value included in the data set for statistical analyses. Standard reference material (SRM) NIES 13 (human hair) was used to assess analytical accuracy; mean SRM recoveries (% recovery ± % RSD) based on 17 replicates over five analytical runs averaged 101 ± 10 for Pb and 98 ± 6 for Cd (both certified values), and 75 ± 14 for Mn and 98 ± 9 for As (both non-certified reference values).

### 2.3. Data Analyses

Summary data are expressed as median or mean ± standard error (SE), or mean ± standard deviation (SD). If necessary, data were square root- or log-transformed to achieve normality and variance equality. To examine within and between subject variation in hair metal levels, data were analyzed using mixed models with metal concentration as dependent variable, hair segment as independent variable, and a repeated statement identifying subject (within subject referring to separate segments of the same strand of hair). Models were adjusted for subject age (continuous) and season of collection, with four seasonal periods (August 2009–October 2009; November 2009–February 2010; March 2010–May 2010; June 2010–August 2010, selected to reflect different climatological seasons that were balanced by the number of subjects). A within-subject effect of hair segment on metal concentration was identified by a significant Type 3 effect. To determine whether the within-subject variation differed by tertile of hair metal concentration, we stratified models by tertile of hair metal concentrations and calculated least square means with a Tukey adjustment. We qualitatively examined the upper and lower confidence bounds of the difference in hair metal levels across tertiles. If the difference and bounds were comparable across tertiles then we concluded that the segment effect on hair metal levels did not differ by tertile. Lastly, to assess whether the segment effect differed by season we added a segment–season interaction term to the unstratified models and identified differences by a significant Type 3 effect. A *p*-value ≤ 0.05 for the various outcomes was considered statistically significant. All data were analyzed using SAS (Version 14.1) or JMP (Version 13.0) software (SAS Institute Inc., Cary, NC, USA, 2016).

## 3. Results

### 3.1. Hair Metal Levels in Children Vary by Several Orders of Magnitude between Subjects and Were Highly Correlated

**Figure 1:**
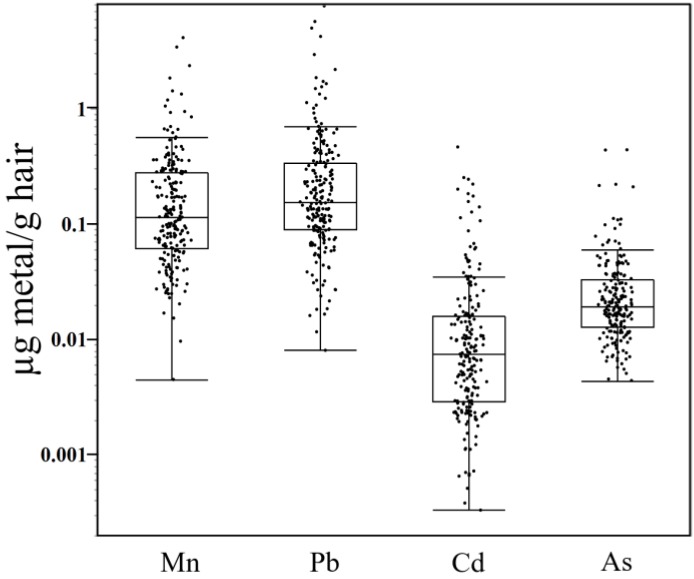
Hair metal concentrations (µg/g, note log scale) in the proximal 2 cm hair segment for male (*n* = 53) and female (*n* = 169) subjects. The horizontal line within the box represents the median, while the upper and lower margins of the boxes represent the 75th and 25th percentiles; whiskers are drawn to the furthest data point within 1.5-times the interquartile range. *N* = 214–222; hair metal values below the limit of detection are excluded.

In this study of 222 subjects (53 male, 169 female), median levels of Mn and Pb in the proximal segment of children’s hair were comparable at 0.109 µg/g (range 0.005–4.10 µg/g) and 0.152 µg/g (range 0.008–7.73 µg/g), respectively (Table 1, Figure 1). Hair levels of Cd and As were both about 10-fold lower than Mn and Pb levels, with median Cd levels of 0.007 µg/g (range 0.0005–0.463) and median As levels of 0.018 µg/g (range 0.004–0.438). Notably, for all four metals there was a ~>100-fold range in proximal segment metal levels between subjects, suggesting substantial differences in the exposure levels and/or incorporation of metal levels into hair between subjects. Moreover, levels of all four metals were higher in the proximal segment of males compared to the proximal segment of females (*p*’s < 0.0001), based on mixed model analysis on log10 transformed data with sex (fixed) and subject (random) factors (*n* = 157 female, 45–53 male, depending on metal). The large majority of hair samples possessed metal levels that were above the analytical detection limits (>97% for Mn, Pb, and Cd, 92% for As).

Associations between the levels of different metals in hair could suggest similar exposure sources. Spearman’s correlation analysis of metal levels in all hair samples of male (proximal segment) and female (proximal, medial, and distal segments) subjects shows that all four metals are highly correlated (*p* < 0.0001), with the correlation between Cd and Pb being strongest (Spearman’s *ρ* = 0.5780), followed by the correlation between Mn and Cd (*ρ* = 0.4475), and Mn and Pb (*ρ* = 0.3513). Correlations between As and the other three metals were weaker (*ρ* < 0.21) (Table 2). 

### 3.2. Variance in Hair Metal Levels between-Subjects is Much Greater than within-Subjects, and Hair Mn, Pb, and Cd, but Not As Concentrations Increase from Proximal to Distal Segments

To determine whether metal levels in hair segments grown over a period of one to two months were more variable between subjects than between adjacent hair segments within a subject, hair samples from female subjects, who typically provided hair samples sufficiently long for segmentation, were cut into sequential two centimeter segments (designated proximal, medial, and distal segments relative to the scalp) for analyses; each two centimeter segment is assumed to reflect roughly two months of hair growth and metal exposure [18,19]. Variance component analysis was used to determine the contribution of subject and hair segment (as variance components) to the variation in hair metal levels between and within subjects. The subject variance component accounted for 65–73% of the variance in hair metal levels, and was substantially greater than the within-subject (i.e., hair segment) variance component, which accounted for 0.1–4% of the variance in hair Mn, Pb, and As, and 12% of the variance in Cd (Table 3). The remainder of the variance in hair metal levels (i.e., 17–35%) was accounted for by the subject–hair segment interaction (Table 3). 

**Figure 2:**
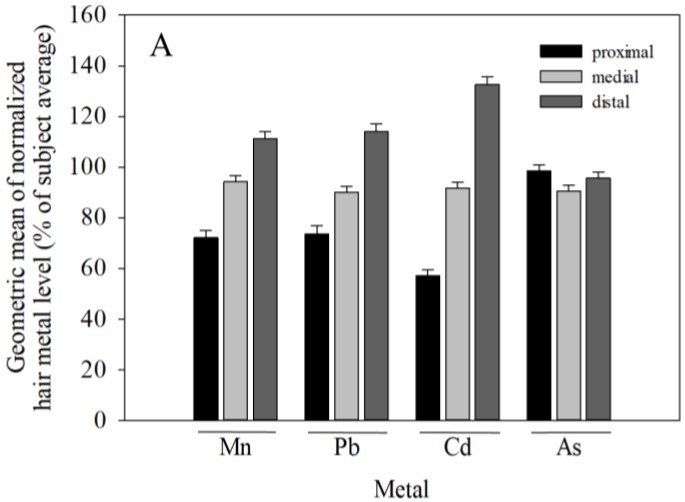

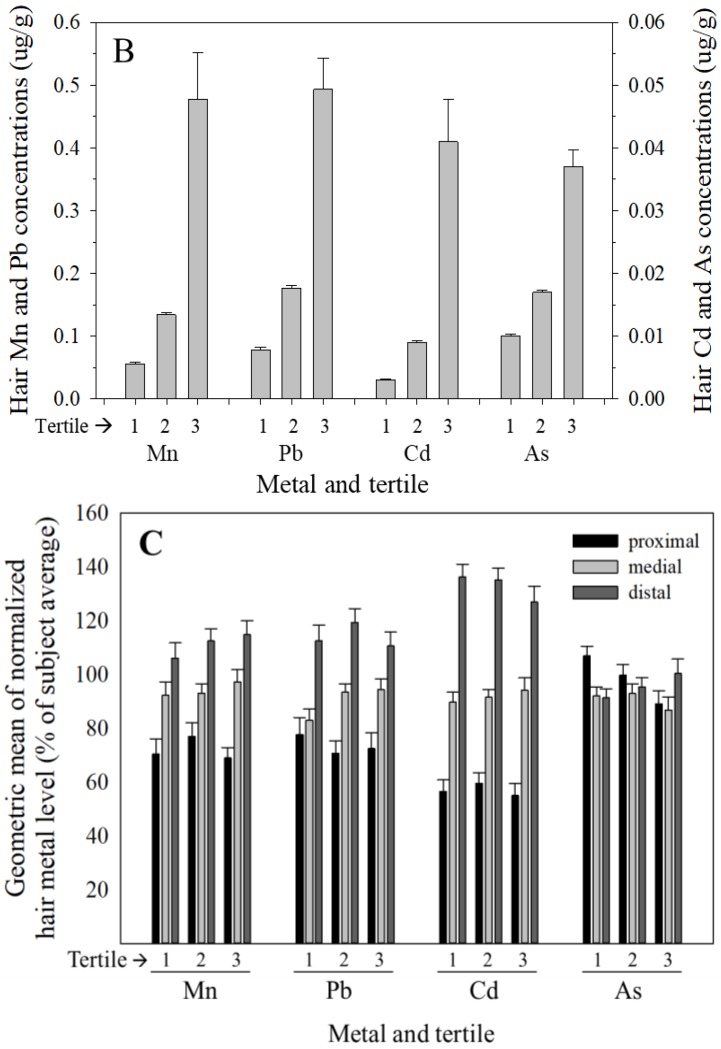
(**A**) Normalized hair Mn, Pb, and Cd, but not As levels systematically increase from proximal to distal two centimeter hair segments from female subjects. Normalized hair metal levels (%) for each segment were calculated by dividing the hair segment metal concentration by the average of all three segments (proximal, medial, distal) for each individual subject. Data are geometric mean (±SE) for all female subjects (*n* = 153–155 per segment and metal); (**B**) Hair Mn, Pb, Cd, and As concentrations differ by ≥5-fold between the lowest and highest tertiles of hair metal levels. Data are mean (±SE) three-segment average of female subjects segregated into tertiles (*n* = 56–57 per tertile and metal); (**C**) The relative increase in hair Mn, Pb, and Cd levels from proximal to distal segments is comparable for subjects with lower (first tertile) versus higher (third tertile) hair metal levels (see text for details).

Given the modest contribution of the hair segment (i.e., within-subject) factor to the variability in hair metal levels in female subjects (i.e., 4–12% for Mn, Pb, and Cd), data for the proximal, medial, and distal two centimeter hair segments of female subjects were analyzed to determine if the metal concentrations *systematically* varied between segments of hair within subjects. To facilitate this, since hair metal concentrations varied substantially between subjects, hair metal concentrations for each segment were expressed as a percentage of the average of the three segments for each female subject, and the geometric mean across subjects of the normalized (%) value for each segment was then calculated for the subjects. Results show that Mn, Pb, and Cd, but not As hair metal concentrations systematically *increased* from the proximal to distal segment (Figure 2a). The relative increase in metal levels from proximal to medial to distal segments was comparable for Mn (72%, 94%, 111% of the three-segment average, respectively) and Pb (74%, 90%, 114% of the three-segment average), and slightly greater for Cd (57%, 92%, 133% of the three-segment average). In contrast, As concentrations were relatively invariant across the proximal, medial, and distal hair segments (98%, 91%, 96% of the three-segment average, respectively) (Figure 2a). Consistent with this, in mixed model analysis with a repeated statement identifying subject, a Type 3 test for fixed effects revealed a significant effect of hair segment on Mn, Pb, and Cd levels (*p* < 0.001, *p* = 0.0015, and *p* < 0.001, respectively), but no significant variation between hair segments in As concentration (*p* = 0.658).

To explore whether this systematic relative increase in hair Mn, Pb, and Cd levels from proximal to distal segments was comparable for subjects with low versus high hair metal levels, we stratified subjects into tertiles based on their three-segment average hair metal concentrations and performed mixed model analysis with a repeated statement identifying subject, as above. For perspective, the systematic ~40–70% relative increase in hair Mn, Pb, and Cd concentrations from the proximal to distal hair segments noted above, while significant, is small compared to the ~100–1000-fold difference in hair metal levels between subjects (Table 1), or the ≥5-fold difference in mean hair metal levels of the lowest and highest tertiles of hair metal levels (Figure 2b). Interestingly, mixed model results show that the relative increase in metal levels from proximal to distal hair segments does not vary by tertile of hair metal concentration for any of the metals (*p*’s = 0.2–>0.9). To visualize this, we stratified subjects into tertiles by their three-segment average hair metal concentrations as above, and normalized the metal concentration for each segment to the three-segment average per subject (expressed as a percent), and then calculated the geometric mean for these normalized (%) values for each tertile of hair metal concentrations. These data reflect the mixed model null results noted above by showing that the *relative* increase in hair Mn, Pb, and Cd from proximal to distal hair segments within female subjects does not differ by tertile of hair metal levels (Figure 2c). 

### 3.3. Hair Metal Levels Vary Seasonally

To explore whether the season of hair sample collection, as a surrogate of possible seasonal differences in exposure or residual exogenous contamination, could explain the systematic increase in hair Mn, Pb, and Cd concentrations from the proximal to distal hair segments, we performed analysis in which a season factor and a segment–season interaction were added to the mixed-model noted above. The four season intervals of August–October 2009, November 2009–February 2010, March–May 2010, and June–August 2010 were selected to align with climate seasons in the Mid-Ohio Valley and to achieve reasonable balance in the number of female subjects across the four season intervals. Results show a significant effect of season on hair Mn, Pb, and Cd levels (*p*’s < 0.001, 0.023, and <0.001, respectively), but not As (*p* = 0.60). There was no season–segment interaction for Mn or Pb (*p*’s > 0.70), although for Cd the interaction was trending towards significance (*p* = 0.073).

**Figure 3:**
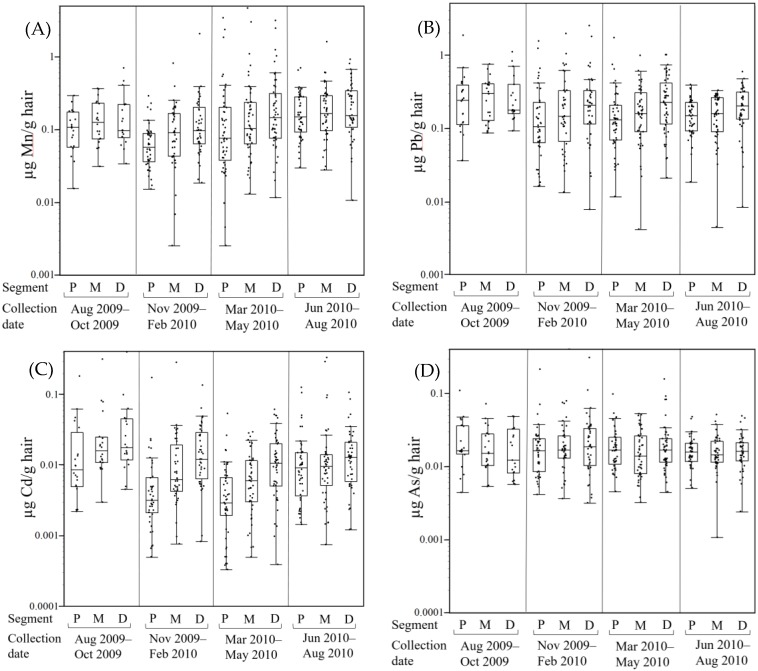
Hair segment Mn (**A**), Pb (**B**), Cd (**C**), and As (**D**) levels in females (µg/g, note log scale) vary with season of collection. Hair metal levels are plotted by three to four months season of collection intervals. The horizontal line within the box represents the median, while the upper and lower margins of the boxes represent the 75th and 25th percentiles; the whiskers are drawn to the furthest data point within 1.5 times the interquartile range. Only female subjects with proximal (P), medial (M), and distal (D) hair segments are shown (*n* = 153–155 subjects per metal).

This effect of season on hair Mn, Pb, and Cd levels is illustrated by generally lower metal concentrations in samples collected in the late fall to spring seasons, and higher concentrations in hair samples collected in summer to early fall seasons (Figure 3). For example, the lowest hair Mn, Pb, and Cd concentrations (seasonal medians of 0.081, 0.146, and 0.0059 µg/g, respectively; all three segments per subject combined) were for samples collected in the November–February (Mn and Pb) or March–May (Cd) seasons. In contrast, the highest hair Mn, Pb, and Cd levels (seasonal medians of 0.157, 0.235, and 0.0142 µg/g, respectively) were for samples collected in the June–August (Mn) or August–October (Pb, Cd) seasons. Across subjects, median levels of hair Mn increased by ~90% between the two seasons, whereas Pb increased by ~60% and Cd by ~240%.

We similarly performed mixed model analysis restricted to metal levels in the proximal segments of males and females, with sex and season of collection as fixed effects and subject as a random effect. Results show a significant effect of sex (*p*’s < 0.0001) and season (*p*’s ≤ 0.004) on proximal segment Mn, Pb, and Cd levels, with higher metal levels in males and higher levels in hair collected in summer/early fall versus winter/spring. For proximal segment As levels, there was a significant effect of sex (*p* < 0.0001, males higher), but no effect of season of hair collection (*p* = 0.137). There was no sex–season interaction for any of the metals (*p*’s > 0.43), indicating that the season of collection did not differently affect male and female hair metal levels.

## 4. Discussion

Hair offers several advantages over other biological tissues/matrices as an exposure biomarker, most notably the potential to retrospectively reconstruct exposures over sequential integrated periods of weeks to months, depending on the length of hair. Here we report Mn, Pb, Cd, and As levels in hair samples from male and female children/adolescents age 6–12 years living in the Mid-Ohio Valley, a region noted for its industrial activity, including the longest operating ferromanganese refinery in North America in Marietta, OH (Eramet Marietta, Inc., Marietta, OH, USA) [2]. This study is unique in that hair samples from females (*n* = 159) were divided into sequential two centimeter segments, and all samples were cleaned prior to analyses using a rigorous cleaning method previously shown to effectively remove exogenous metal contamination [14]. 

### 4.1. Correlations between Metals Suggests Some Shared Exposure Sources/Pathways

The significant correlations among hair levels of all four metals, with correlations of hair Mn, Pb, and Cd being strongest (Spearman’s *ρ*’s ~0.35–0.58 for all hair samples, Table 2) suggests some shared environmental sources/pathways for the incorporation of these metals into hair, and possibly also similar chemistries of interaction of several of these metals with hair keratin. The Mid-Ohio valley region has a history of industrial activity, including ferromanganese alloy and perfluoroocatnoate (PFOA) chemical manufacturing, and air monitoring in 2007–2008 measured levels of Cd, As, and particularly Mn that exceeded ATSDR and EPA health-based comparison values [29]. Further, human hair is a complex biological matrix composed predominantly of proteins (65–95%), water (up to 32% by weight depending on its moisture content), lipids, pigment, and trace elements that are coordinated with the functional groups of protein amino acids or with fatty-acid groups of lipids [19]. Though the protein composition of hair may vary across individuals, it is generally rich in polar and charged amino acids, including hydroxyls, amides, acidic and basic amino acids, and disulfides, and these are the components of hair that may readily coordinate with endogenously and exogenously incorporated metals [19,30]. Thus, the stronger correlations between hair Pb and Cd could also reflect similar chemistries of interaction of these two metals with hair keratin; Cd and Pb have similar affinities to sulfur and nitrogen ligands, while Mn coordinates strongly with oxygen ligands [19,30]. Since As is assumed to exist mainly as oxyanion species, it likely chemically coordinates with different functional groups in hair keratin fibers than the cationic metals. 

### 4.2. Hair Metal Levels Vary Substantially More between Subjects than within Subjects, and Hair Mn, Pb, and Cd, but Not As Concentrations Increase from Proximal to Distal Segments

We found that hair Mn, Pb, Cd, and As levels were higher in males compared to females, and that levels varied by ~100–1000-fold between all subjects (Table 1, Figure 1), but varied comparatively little within subjects, with relative changes of ~40–60% in hair Mn, Pb, and Cd, and <10% in As levels across the proximal, medial, and distal hair segments of female subjects (Figure 3). Consistent with this, variance component analysis showed that the between subject factor accounted for the majority (65–73%) of variance in hair metal levels, while the within subject component (i.e., variation between hair segments within female subjects) accounted for ~4% of the variance in Mn and Pb, 12% in Cd, but very little of the within subject variance in As (0.1%). Together, these findings suggest that hair metal levels reflect important between subject differences in metal exposure and incorporation of metals into hair.

Notably, concentrations of hair Mn, Pb, and Cd, but not As systematically increased from the proximal to distal two centimeter hair segment in female subjects. Since hair samples were collected from subjects throughout the Mid-Ohio Valley over a 13 months period, it is unlikely that this increase in metal concentrations from proximal to distal hair segments reflects temporal differences in metal exposure common to all subjects. Alternatively, we considered whether residual exogenous metal contamination that remained after rigorous cleaning could account for the increase in hair metal levels across segments. Given that subjects likely inhabited environments with inherently different environmental exposure burdens, as suggested by the 100–1000-fold difference in hair metal levels between subjects, we reasoned that subjects in higher metal exposure environments would have experienced both higher endogenous metal exposures, leading to greater metal incorporation into growing hair, and higher exogenous metal contamination of hair compared to subjects living in lower metal burden environments. Moreover, we considered that the older, distal hair segments likely acquired more exogenous metal contamination than the younger proximal segments, because the older distal segments were in contact with the environment roughly four months longer than the proximal segments, and thus were exposed to a greater cumulative environmental exposure burden. Following this logic with the assumption that environmental exposures from water, air, dust, etc. are likely the primary exposure source(s) for both the endogenous and exogenous components of hair metal levels, the *relative* contribution of residual exogenous contamination to total hair metal levels would scale with (i) the magnitude of environmental metal contamination and the duration of time the hair was in contact with the environment, and (ii) the endogenous (metabolically incorporated) component of hair metal levels. In this case the *relative* (percent) increase in hair Mn, Pb, and Cd levels from the proximal to distal hair segments would be comparable for subjects in the lowest and highest tertiles of hair metal levels, which is consistent with our findings (Figure 2c). Collectively, these findings further suggest that the proximal segment hair metal levels reflect predominantly endogenously-incorporated metals, while distal segment metal levels reflect both endogenously-incorporated metals and a relatively small but notable exogenously-added (contamination) component of hair metals (i.e., Mn, Pb, and Cd), the latter in spite of the rigorous hair cleaning methodology used here [14]. 

Skröder et al. [17] similarly reported systematic increases in hair Mn, Pb, and Cd, but not As in sequential hair segments over eight-centimeter of hair length in a small number of Bangladeshi children (*n* = 19). In that study the relative increase in hair metal levels with distance from the scalp was 4.6-fold for Mn, and roughly two to three-fold for Pb and Cd-relative increases that are much greater than the ~40–70% relative increase from proximal to distal hair segments observed in the present study. Skröder et al. interpreted their findings as evidence of exogenous metal contamination that was most pronounced for hair Mn levels. In light of (i) the very elevated groundwater Mn levels in the Bangladeshi subjects’ environment; and (ii) our studies showing that hair is readily and significantly contaminated from direct contact with Mn-contaminated water and that exogenous Mn contamination from water is incompletely removed even with rigorous cleaning [14], it is likely that the hair Mn levels reported by Skröder et al. are dominated by unresolved exogenous contamination.

### 4.3. Hair Metal Levels Vary by Season of Collection

We found that the season of hair collection was associated with hair levels of Mn, Pb, and Cd, but not As, and that there was no season–sex (proximal segments only) or season–hair segment (females only) interaction in the mixed model analyses. This suggests that the main effect of season of hair collection may reflect a contribution of seasonal differences in residual exogenous metal contamination that slightly but measurably contributed to hair metal levels. Since hair samples were collected over a 13 month period, the proximal and distal hair segments of females would have grown over different seasons depending on the season of hair collection [18,19]. Male and female subjects whose hair was collected in late summer/early fall, when outdoor activity and some routes of exposure might be greatest, had higher levels of Mn, Cd, and Pb in proximal hair segments (males and females) and across all three segments of females, compared to hair samples collected in winter and spring. If hair contained only endogenously-incorporated metals, then seasonal differences in exposure would be associated with the season of hair growth, not the season of hair collection as observed. 

### 4.4. Hair Metal Concentrations Reported Here Are Generally Lower than other Studies in Children

To facilitate inter-study comparison of reported hair metal levels in children, we summarized reported findings from 15 studies of similarly aged children (Table 4). Median or mean hair Mn levels differ by ~120-fold across studies, while hair levels of Pb, Cd, and As differ across studies by ~15-fold, 4-fold, and 180-fold, respectively. These differences between studies may reflect, at least in part, differences in endogenous metal exposure and incorporation of metals from the circulation into hair. However, because of the susceptibility of hair to environmental metal contamination, differences in hair metal levels between studies likely also reflect differences in unresolved exogenous contamination. The listed studies used a variety of different hair cleaning methodologies, from no cleaning to multi-stage cleaning procedures employing various combinations of detergents and/or solvents, weak acid, and sonication (Table 4). For example, Skröder et al. [17] reported median hair Mn levels of 5.0 µg/g in Bangladeshi children exposed to elevated Mn in drinking water, while Hernandez-Bonilla et al. [22] and Menezes-Filho et al. [21] reported hair Mn levels greater than 10 µg/g in Mexican and Brazilian children, respectively, living in the vicinity of ferromanganese alloy plants. These hair Mn levels are nearly two orders of magnitude or more higher than levels reported in the present study, or levels reported by Torrente et al. [31] and Lucas et al. [12] for Spanish and Italian children, respectively, living in areas impacted by industrial emissions. Skröder et al. [17] and Hernandez-Bonilla et al. [22] reported cleaning hair prior to analysis with a Triton detergent wash, while Menezes-Filho et al. [21] and Torrente et al. [31] used a Triton wash with ultrasound sonication. The present study and Lucas et al. [12] used Triton sonication followed by sonication in a 1 N nitric acid solution. While it is difficult to separate the influence of environmental exposure from the efficacy of hair cleaning methods to reduce exogenous hair contamination, these data suggest that studies that used more rigorous cleaning procedures reported lower hair Mn (and generally other metals) concentrations, consistent with studies showing that the rigor of hair cleaning prior to analyses significantly influences hair metal levels from exogenous contamination [14,20,26]. 

There are several studies from different geographical regions that used the same cleaning method, as well as studies from the same geographical region that used different cleaning methods that can be readily compared to estimate the extent that differences in hair metal levels between studies reflect differences in exposure versus differences in exogenous contamination due to different hair cleaning methods. For example, prior studies from our lab in Italian children exposed to environmental metals from industrial ferroalloy emissions [12,14] used the same Triton sonication followed by dilute nitric acid sonication hair cleaning method as the present study. Hair Mn and Pb levels are very comparable between these studies (Table 4), allowing us to conclude that the subjects in the present study had ~10–30% higher Mn exposure and ~10% lower Pb exposure levels compared to the Italian subjects, based on hair metal levels. We can also compare hair Mn levels between two studies from the same region that used different hair cleaning methods. Haynes et al. [2] used a Triton detergent hair cleaning method (without sonication) and reported geometric mean hair Mn levels of 0.417 µg/g from children in the same Ohio Valley region as the present study, which are greater than three-fold higher than levels in the present study (geometric mean Mn of 0.119 µg/g, median 0.109 µg/g, Table 4). This difference in hair Mn levels between the two studies may be due to differences in Mn exposure between cohorts. However, it may also be that they are due to differences in hair cleaning methods, given that our prior study [14] found that a Triton sonication hair cleaning method similar to that used by Haynes et al. [2] yielded hair Mn and Pb levels that were ~2.5–4-fold higher than hair metal levels following the Triton + weak nitric acid sonication method used in the present study. Collectively, these findings suggest that the extent that hair metal levels reflect endogenous exposure will vary substantially depending on the hair cleaning method and the extent that cleaning reduces exogenous hair contamination, the latter of which may also vary between subjects and hair type [14,26]. 

### 4.5. Hair Metal Levels as a Biomarker of Exposure and Associated Health Effects

Studies have reported mixed results regarding the extent that hair metal levels are associated with metal levels in environmental media (e.g., water, soil, dust, and airborne particles, etc.), or biomarkers of endogenous metal exposure (blood, urine, nails). Lucas et al. [12] reported low but statistically significant correlations between children’s hair Mn and Mn levels in household dust (*ρ*’s ~ 0.27, *p* < 0.001) and airborne particles (*ρ* = 0.126, *p* < 0.05). Bouchard et al. [13] and Oulhote et al. [23] reported that hair Mn levels were higher in Canadian children exposed to Mn-contaminated water compared to children living in homes with a private well with lower water Mn levels, while Skröder et al. [17] reported no correlation between Mn levels in water and hair Mn levels in Bangladeshi children. 

Studies have also reported mixed results on the associations between metal levels in hair and other exposure biomarkers. Hair As levels have been shown to reflect the internal body burden of As, based on strong correlations between hair As levels with As levels in erythrocytes (*ρ* = 0.73, *p* < 0.001) and urine (*ρ* = 0.66, *p* < 0.001) [17], while hair Cd and Pb levels are not generally recognized as reliable predictors of exposure and internal dose [7,11,17]. A number of studies have reported no association between hair Mn and Mn levels in blood [2,12,38] or erythrocytes [17], while others reported low but significant correlations between hair and blood Mn levels [22,39], and associations between hair Mn and Mn levels in fingernails (*ρ* = 0.247, *p* < 0.001) [12]. In their recent study, Skröder et al. [17] concluded that levels of Mn in hair do not reflect the actual internal Mn dose in Bangladeshi children, but the authors acknowledged that their findings strongly pointed to significant external contamination of hair from Mn-contaminated water, which would preclude the ability to even test whether hair Mn reflects the internal Mn burden. Finally, there is substantial evidence showing the hair Mn levels are associated with a number of neurodevelopmental health effects, including reduced IQ, learning, memory, and perceptual reasoning, and greater hyperactive and oppositional behaviors [2,13,23,38,39,40,41], leading Coetzee et al. [3] to conclude in their recent review that hair was the most consistent and valid biomarker of manganese exposure and associated neurodevelopmental health effects in children. 

The present study had several limitations. First, the parent C8 Health Project Neurobehavioral Development Follow-up study, which was investigating the neurodevelopmental health effects of perfluoroocatnoate (PFOA) exposure in southeastern Ohio and northwestern West Virginia [27], did not assess metal exposures in the subjects’ environment (e.g., air, dust, water) or in other biomarker tissues (e.g., blood), thereby limiting our ability to interpret the hair metal levels reported here as a biomarker of environmental metal exposures or internalized body burden. Second, sequential hair segments were available only for female subjects, and not males, precluding assessment of a sex–hair segment interaction in our statistical models. 

## Figures and Tables

**Table 1:** Hair metal concentrations (µg metal/g hair) of two centimeter hair segments from males and females *.

Sex	Metal	Segment	N	Mean	Median	Range	S.D.
Males	Mn	proximal	53	0.406	0.232	0.0248–4.10	0.616
	Pb		52	0.829	0.414	0.0415–7.73	1.38
	Cd		51	0.0540	0.0188	0.0039–0.463	0.0861
	As		45	0.0630	0.0366	0.0061–0.438	0.0893
Females	Mn	proximal	169	0.184	0.0948	0.0045–3.40	0.356
		medial	157	0.226	0.126	0.0067–4.63	0.463
		distal	155	0.253	0.135	0.0184–3.12	0.388
	Pb	proximal	169	0.242	0.134	0.0080–5.66	0.493
		medial	156	0.229	0.163	0.0042–1.95	0.229
		distal	153	0.284	0.208	0.0078–2.49	0.298
	Cd	proximal	169	0.0119	0.0050	0.0005–0.182	0.0242
		medial	157	0.0202	0.0088	0.0005–0.335	0.0491
		distal	155	0.0215	0.0128	0.0004–0.401	0.0369
	As	proximal	169	0.0227	0.0165	0.0044–0.214	0.0259
		medial	157	0.0197	0.0155	0.0011–0.0789	0.0143
		distal	155	0.0238	0.0170	0.0024–0.310	0.0307

* The reported ‘*n*’ and the ranges in metal levels are for measured values above the limit of detection (see text).

**Table 2:** Spearman’s correlation (*n* = number of hair samples) between hair metal levels in all hair samples from males (proximal segment) and females (proximal, medial, and distal segments); all Spearman’s *ρ* values are highly significant, *p* < 0.0001).

	Mn		
**Pb**	0.3513 (530)	**Pb**	
**Cd**	0.4475 (532)	0.5780 (529)	**Cd**
**As**	0.1872 (526)	0.1814 (523)	0.2081 (526)

**Table 3:** Percent of variance in hair metal levels attributed to between-subject, within-subject (i.e., hair segment), and subject–segment interaction components according to interclass correlation analysis.

Component	Mn	Pb	Cd	As
**Between-subject**	72.8	71.5	70.3	65.1
**Within-subject**	4.1	3.8	12.4	0.1
**Interaction**	23.1	24.7	17.3	34.8

**Table 4:** Comparison of hair metal concentrations in children from the present study with levels reported in children (ages 4–14) in other studies that used a variety of different hair cleaning methods prior to analysis.

Location (Study) *	Cleaning Method ^#^	Sub-Population (N) ^&^	Mn µg/g	Pb µg/g	Cd µg/g	As µg/g
U.S., Ohio (this study) ^a^	T/S, N/S	all (222)	0.109 (0.441)	0.152 (0.829)	0.007 (0.050)	0.018 (0.049)
Italy [12], ^a^	T/S, N/S	all (501)	0.098 (0.139)	NA	NA	NA
Spain [32], ^b^	T/S, E/S	males (96)	NA	NA	0.003 (0.003-0.004)	NA
		females (124)	NA	NA	0.006 (0.004-0.007)	NA
		combined (220)	0.137 (NA)	0.14 (NA)	NA	0.017 (NA)
Brazil [21], ^a^	T/S	exposed males (34)	12.1 (9.9)	NA	NA	NA
		exposed females (36)	12.4 (13.4)	NA	NA	NA
Brazil [33], ^a^	T/S	referents (44)	NA	2.09 (2.06)	NA	NA
		exposed (88)	NA	1.26 (3.70)	NA	NA
Spain [31], ^c^	T/S	urban area (45)	0.26 (0.90)	0.32 (0.30)	<0.03	NA
		industrial area (54)	0.18 (0.28)	1.59 (3.01)	<0.03	NA
Tibet [34], ^b^	T	exposed (22)	4.28 (5.36)	NA	NA	NA
		unexposed 1 (24)	2.87 (3.05)	NA	NA	NA
		unexposed 2 (24)	2.44 (3.00)	NA	NA	NA
Bangladesh [17], ^d^	T	all (207)	5.0 (1.4–23)	1.6 (0.50–6.4)	0.029 (0.0008–0.150)	0.53 (0.14–2.9)
U.S., Ohio [2], ^b^	T	all (370)	0.417 (0.002)	NA	NA	NA
Greece [35], ^b^	T	urban area 1 (11)	NA	0.78 (1.47)	0.014 (0.028)	0.020 (0.029)
		urban area 2 (21)	NA	1.29 (6.86)	0.023 (0.021)	0.036 (0.011)
		suburban area (19)	NA	0.60 (0.67)	0.015 (0.037)	0.026 (0.009)
Mexico [22], ^b^	T	unexposed (93)	0.57 (0.49–0.66)	NA	NA	NA
		exposed (79)	12 (10.7–13.8)	NA	NA	NA
Spain [36], ^d^	A/S	all (648)	0.33 (0.12–0.94)	0.70 (0.17–4.28)	0.018 (0.004–0.079)	0.07 (<0.05–0.26)
Italy [24], ^a^	A/S	males (130)	0.27 (0.25)	0.78 (0.76)	0.03 (0.05)	<0.01
		females (94)	0.31 (0.27)	0.79 (0.80)	0.03 (0.05)	<0.01
Russia [37], ^a^	A	unexposed (84)	2.25 (3.77)	1.55 (2.98)	0.12 (0.18)	0.030 (0.034)
		exposed (82)	1.60 (3.51)	2.48 (4.20)	0.11 (0.14)	0.020 (0.042)
Vietnam [25], ^c^	A	control males (5)	NA	NA	NA	0.31 (0.07)
		control females (4)	NA	NA	NA	0.36 (0.17)
		exposed males (22)	NA	NA	NA	2.76 (2.54)
		exposed female (44)	NA	NA	NA	5.59 (7.90)
Quebec [23], ^b^	U	males (148)	0.75 (NA)	NA	NA	NA
		females (164)	0.8 (NA)	NA	NA	NA

* Study citation number and data type; ^a^ = data are median (SD); ^b^ = data are geometric mean (SD, geometric SD, or 95% CI); ^c^ = data are mean (SD); ^d^ = data are median (5th–95th percentile). NA = not analyzed or reported. ^#^ Hair cleaning methods, T = Triton, N = nitric acid, /S = sonicated, E = ethanol, A = acetone, U = uncleaned. ^&^ Study sub-population by gender and study sub-site location when reported (all = both males and females across study sub-sites).

## References

[1] Smith D., Gwiazda R., Bowler R., Roels H., Park R., Taicher C., Lucchini R. (2007). Biomarkers of Mn Exposure in Humans. Am. J. Ind. Med..

[2] Haynes E.N., Sucharew H., Kuhnell P., Alden J., Barnas M., Wright R.O., Parsons P.J., Aldous K.M., Praamsma M.L., Beidler C. (2015). Manganese Exposure and Neurocognitive Outcomes in Rural School-Age Children: The Communities Actively Researching Exposure Study (Ohio, USA). Environ. Health Perspect..

[3] Coetzee D.J., McGovern P.M., Rao R., Harnack L.J., Georgieff M.K., Stepanov I. (2016). Measuring the Impact of Manganese Exposure on Children’s Neurodevelopment: Advances and Research Gaps in Biomarker-Based Approaches. Environ. Health.

[4] Arora M., Austin C., Sarrafpour B., Hernańdez-Ávila M., Hu H., Wright R.O., Tellez-Rojo M.M. (2014). Determining Prenatal, Early Childhood and Cumulative Long-Term Lead Exposure Using Micro-Spatial Deciduous Dentine Levels. PLoS ONE.

[5] Sanders A.P., Claus Henn B., Wright R.O. (2015). Perinatal and Childhood Exposure to Cadmium, Manganese, and Metal Mixtures and Effects on Cognition and Behavior: A Review of Recent Literature. Curr. Environ. Health Rep..

[6] Zoni S., Lucchini R.G. (2013). Manganese Exposure: Cognitive, Motor and Behavioral Effects on Children: A Review of Recent Findings. Curr. Opin. Pediatr..

[7] Bergdahl I.A., Skerfving S. (2008). Biomonitoring of Lead Exposure—Alternatives to Blood. J. Toxicol. Environ. Health Part A.

[8] Fowler B.A. (2009). Monitoring of Human Populations for Early Markers of Cadmium Toxicity: A Review. Toxicol. Appl. Pharmacol..

[9] Roels H.A., Hoet P., Lison D. (1999). Usefulness of Biomarkers of Exposure to Inorganic Mercury, Lead, or Cadmium in Controlling Occupational and Environmental Risks of Nephrotoxicity. Ren. Fail..

[10] Lanphear B.P., Hornung R., Khoury J., Yolton K., Baghurst P., Bellinger D.C., Canfield R.L., Dietrich K.N., Bornschein R., Greene T. (2005). Low-Level Environmental Lead Exposure and Children’s Intellectual Function: An International Pooled Analysis. Environ. Health Perspect..

[11] (1993). Measuring Lead Exposure in Infants, Children, and other Sensitive Populations.

[12] Lucas E.L., Bertrand P., Guazzetti S., Donna F., Peli M., Jursa T.P., Lucchini R., Smith D.R. (2015). Impact of Ferromanganese Alloy Plants on Household Dust Manganese Levels: Implications for Childhood Exposure. Environ. Res..

[13] Bouchard M.F., Sauvé S., Barbeau B., Legrand M., Brodeur M.È., Bouffard T., Limoges E., Bellinger D.C., Mergler D. (2011). Intellectual Impairment in School-Age Children Exposed to Manganese from Drinking Water. Environ. Health Perspect..

[14] Eastman R.R., Jursa T.P., Benedetti C., Lucchini R.G., Smith D.R. (2013). Hair as a Biomarker of Environmental Manganese Exposure. Environ. Sci. Technol..

[15] Crinella F.M. (2012). Does Soy-Based Infant Formula Cause ADHD? Update and Public Policy Considerations. Expert Rev. Neurother..

[16] Branco V., Caito S., Farina M., Teixeira da Rocha J., Aschner M., Carvalho C. (2017). Biomarkers of Mercury Toxicity: Past, Present, and Future Trends. J. Toxicol. Environ. Health Part B.

[17] Skröder H., Kippler M., Nermell B., Tofail F., Levi M., Rahman S.M., Raqib R., Vahter M. (2017). Major Limitations in Using Element Concentrations in Hair as Biomarkers of Exposure to Toxic and Essential Trace Elements in Children. Environ. Health Perspect..

[18] Harkey M.R. (1993). Anatomy and Physiology of Hair. Forensic Sci. Int..

[19] Robbins C.R. (2012). Chemical and Physical Behavior of Human Hair.

[20] Razagui I.B.-A. (2008). A Comparative Evaluation of Three Washing Procedures for Minimizing Exogenous Trace Element Contamination in Fetal Scalp Hair of Various Obstetric Outcomes. Biol. Trace Elem. Res..

[21] Menezes-Filho J.A., de Carvalho-Vivas C.F., Viana G.F.S., Ferreira J.R.D., Nunes L.S., Mergler D., Abreu N. (2014). Elevated Manganese Exposure and School-Aged Children’s Behavior: A Gender-Stratified Analysis. Neurotoxicology.

[22] Hernández-Bonilla D., Schilmann A., Montes S., Rodríguez-Agudelo Y., Rodríguez-Dozal S., Solís-Vivanco R., Ríos C., Riojas-Rodríguez H. (2011). Environmental Exposure to Manganese and Motor Function of Children in Mexico. Neurotoxicology.

[23] Oulhote Y., Mergler D., Barbeau B., Bellinger D.C., Bouffard T., Brodeur M.-È., Saint-Amour D., Legrand M., Sauvé S., Bouchard M.F. (2014). Neurobehavioral Function in School-Age Children Exposed to Manganese in Drinking Water. Environ. Health Perspect..

[24] Dongarrà G., Lombardo M., Tamburo E., Varrica D., Cibella F., Cuttitta G. (2011). Concentration and Reference Interval of Trace Elements in Human Hair from Students Living in Palermo, Sicily (Italy). Environ. Toxicol. Pharmacol..

[25] Hanh H.T., Kim K.-W., Bang S., Hoa N.M. (2011). Community Exposure to Arsenic in the Mekong River Delta, Southern Vietnam. J. Environ. Monit..

[26] Stauber J.L., Florence T.M. (1989). Manganese in Scalp Hair: Problems of Exogenous Manganese and Implications for Manganese Monitoring in Groote Eylandt Aborigines. Sci. Total Environ..

[27] Stein C.R., Savitz D.A., Bellinger D.C. (2013). Perfluorooctanoate and Neuropsychological Outcomes in Children. Epidemiology.

[28] Rugless F., Bhattacharya A., Succop P., Dietrich K.N., Cox C., Alden J., Kuhnell P., Barnas M., Wright R., Parsons P.J. (2014). Childhood Exposure to Manganese and Postural Instability in Children Living near a Ferromanganese Refinery in Southeastern Ohio. Neurotoxicol. Teratol..

[29] Agency for Toxic Substances and Disease Registry (ATSDR) (2009). Health Consultation: Marietta Area Air Investigation Marietta, Ohio.

[30] Da Silva J.J.R.F., Williams R.J.P. (2001). The Biological Chemistry of the Elements: The Inorganic Chemistry of Life.

[31] Torrente M., Colomina M.T., Domingo J.L. (2005). Metal Concentrations in Hair and Cognitive Assessment in an Adolescent Population. Biol. Trace Elem. Res..

[32] Molina-Villalba I., Lacasaña M., Rodríguez-Barranco M., Hernández A.F., Gonzalez-Alzaga B., Aguilar-Garduño C., Gil F. (2015). Biomonitoring of Arsenic, Cadmium, Lead, Manganese and Mercury in Urine and Hair of Children Living near Mining and Industrial Areas. Chemosphere.

[33] Menezes-Filho J.A., de Sousa Viana G.F., Paes C.R. (2012). Determinants of Lead Exposure in Children on the Outskirts of Salvador, Brazil. Environ. Monit. Assess..

[34] Guo Y., Li H., Yang L., Li Y., Wei B., Wang W., Gong H., Guo M., Nima C., Zhao S. (2017). Trace Element Levels in Scalp Hair of School Children in Shigatse, Tibet, an Endemic Area for Kaschin-Beck Disease (KBD). Biol. Trace Elem. Res..

[35] Evrenoglou L., Partsinevelou S.A., Stamatis P., Lazaris A., Patsouris E., Kotampasi C., Nicolopoulou-Stamati P. (2013). Children Exposure to Trace Levels of Heavy Metals at the North Zone of Kifissos River. Sci. Total Environ..

[36] Llorente Ballesteros M.T., Navarro Serrano I., Izquierdo Álvarez S. (2017). Reference Levels of Trace Elements in Hair Samples from Children and Adolescents in Madrid, Spain. J. Trace Elem. Med. Biol..

[37] Drobyshev E.J., Solovyev N.D., Ivanenko N.B., Kombarova M.Y., Ganeev A.A. (2017). Trace Element Biomonitoring in Hair of School Children from a Polluted Area by Sector Field Inductively Coupled Plasma Mass Spectrometry. J. Trace Elem. Med. Biol..

[38] Menezes-Filho J.A., Novaes C.d.O., Moreira J.C., Sarcinelli P.N., Mergler D. (2011). Elevated Manganese and Cognitive Performance in School-Aged Children and Their Mothers. Environ. Res..

[39] Torres-Agustín R., Rodríguez-Agudelo Y., Schilmann A., Solís-Vivanco R., Montes S., Riojas-Rodríguez H., Cortez-Lugo M., Ríos C. (2013). Effect of Environmental Manganese Exposure on Verbal Learning and Memory in Mexican Children. Environ. Res..

[40] Bouchard M., Mergler D., Baldwin M., Panisset M., Bowler R., Roels H.A. (2007). Neurobehavioral Functioning after Cessation of Manganese Exposure: A Follow-up after 14 Years. Am. J. Ind. Med..

[41] Wright R.O., Amarasiriwardena C., Woolf A.D., Jim R., Bellinger D.C. (2006). Neuropsychological Correlates of Hair Arsenic, Manganese, and Cadmium Levels in School-Age Children Residing near a Hazardous Waste Site. Neurotoxicology.

